# Anatomy of protein disorder, flexibility and disease-related mutations

**DOI:** 10.3389/fmolb.2015.00047

**Published:** 2015-08-12

**Authors:** Hui-Chun Lu, Sun Sook Chung, Arianna Fornili, Franca Fraternali

**Affiliations:** ^1^Randall Division of Cell and Molecular Biophysics, King's College LondonLondon, UK; ^2^Department of Haematological Medicine, King's College LondonLondon, UK; ^3^School of Biological and Chemical Sciences, Queen Mary University of LondonLondon, UK

**Keywords:** non-synonymous SNPs, protein disorder, order-disorder propensity, disease-related mutations, protein flexibility

## Abstract

Integration of protein structural information with human genetic variation and pathogenic mutations is essential to understand molecular mechanisms associated with the effects of polymorphisms on protein interactions and cellular processes. We investigate occurrences of non-synonymous SNPs in ordered and disordered protein regions by systematic mapping of common variants and disease-related SNPs onto these regions. We show that common variants accumulate in disordered regions; conversely pathogenic variants are significantly depleted in disordered regions. These different occurrences of pathogenic and common SNPs can be attributed to a negative selection on random mutations in structurally highly constrained regions. New approaches in the study of quantitative effects of pathogenic-related mutations should effectively account for all the possible contexts and relative functional constraints in which the sequence variation occurs.

## Introduction

Because of the intrinsic complexity of biological systems, reductionist approaches have traditionally been used that concentrate on carefully chosen sub-systems. The availability of complete genome sequences and large (but incomplete) collections of biomolecular structures at atomic resolution favors large-scale computational approaches to investigate multiple components and their interactions (Lu et al., 2013). The undisputed relationship between protein-coding elements and their protein products has dominated the field of genomics/proteomics research in the past and the relationship between structure and function has been widely investigated.

Large-scale studies have been performed on how disease-related mutations may disrupt protein functions and ultimately regulate the function of biological systems (Studer et al., 2013). Mutations are classified as “loss of function,” “gain of function,” or “neutral” according to their effect on protein function. These effects can be mediated by alterations of the protein stability induced by the mutation (Yue et al., 2005; Studer et al., 2013). The impact of SNPs on protein function and structural stability has been extensively studied at the level of the single protein (Yue et al., 2005; Schuster-Bockler and Bateman, 2008; Wang et al., 2012; Nishi et al., 2013; Studer et al., 2013; Yates and Sternberg, 2013; Scharner et al., 2014) and a number of predictors have been developed to evaluate the impact of SNPs on individual proteins (Thomas and Kejariwal, 2004; Capriotti et al., 2005; Bromberg and Rost, 2007; Adzhubei et al., 2010; Reva et al., 2011; Al-Numair and Martin, 2013; Shihab et al., 2013; Pires et al., 2014; Yates et al., 2014). With the intention to expand the single protein structure-function paradigm, the interplay between Protein Protein Interactions (PPI) networks, structures, and disease mutations has been explored by several groups (see reviews Lu et al., 2013; Yates and Sternberg, 2013) and reference therein, Kelley et al., 2015; Mosca et al., 2015). Particularly the crucial role of interfaces in modulating the effects of pathogenic variation in binding and signaling (Stefl et al., 2013; Yates and Sternberg, 2013) has been generally accepted. In recent years additional findings have contributed to further expanding classical structure-function approaches: firstly, the widely recognized importance of non-coding elements (Necsulea and Kaessmann, 2014; Ling et al., 2015) (not discussed here) and the enrichment of SNPs in these (Consortium, 2012; Kircher et al., 2014); secondly, the role of unstructured regions, intrinsically disordered elements and flexibility in protein function versatility (Uversky, 2013; Dunker et al., 2015; Wright and Dyson, 2015). Even in the absence of intrinsic disorder, there is growing evidence that conformational flexibility is important in regulating protein-protein interactions (Dobbins et al., 2008; Stefl et al., 2013; Uversky, 2013). This effect has also been shown for proteins that have multiple partners (hubs) and are essential in protein-protein communication and signaling. Hubs' promiscuous binding sites have been demonstrated to display specific dynamical properties, pre-existing in the isolated state of the individual protein, allowing for polyvalent partner binding (Fornili et al., 2013). In any case, quantification of the occurrence of SNPs in disordered and flexible protein regions is a complex task, because different shades of disorder have been identified as playing a role in protein function stability and binding (Uversky et al., 2014; Wright and Dyson, 2015 and references therein). One particularly interesting case is represented by mutations related to disorder-to-order (D-O) transitions; there are often associated to post-translational modifications or with defense mechanisms to protect proteins from toxic aggregation and oxidative stress (Winter et al., 2008) and therefore may result in a stronger impact on the protein functional role. Consequently, order/disorder-sensitive descriptors of the specific chemico-physical environment in the vicinity of the observed variant are needed to evaluate rigorously the relationship between disorder and disease-related mutations.

We aim to contribute to this debate by exploring and quantifying in a systematic way the relationship between order/disorder and the occurrence of common variants (dbSNP: common variations from the 1000 Genomes project, Sherry et al., 2001), disease-related SNPs (OMIM: Mendelian genetic diseases, Hamosh et al., 2005) and cancer-related SNPs (COSMIC, Forbes et al., 2011). To this end we decompose the protein sequences anatomically in folded domain regions, unfolded-disordered (intra-domain) regions and inter-domain disordered regions and calculate the enrichment/depletion of SNPs in each of these regions. These comparisons based on mapping SNPs on static, crystallographic structures, represent a first step in quantifying the different roles played by the two (ordered vs. disordered) environments in which a common or pathogenic mutation may occur. We also explored scenarios of mutual effects of mutations in ordered regions on the disorder content within that domain. We discuss two cases of hubs that are strongly involved in cancer: BRAF (Haling et al., 2014; Thevakumaran et al., 2015) and JAK2 (Bandaranayake et al., 2012), both with phenotypic pathogenic mutations occurring in ordered regions and affecting the disorder content of distal sites in the domain.

## Results

### Dearth of disease-related SNPs in inter-domain disordered regions

The relative enrichment of SNPs in the dissected disordered regions of the protein have been analyzed by comparing three different classes of SNPs: (a) the common variants from the 1000 Genomes project (dbSNP) (Sherry et al., 2001), (b) the genetic-disease variants from OMIM (Hamosh et al., 2005), and (c) the COSMIC cancer-related SNPs (Forbes et al., 2011). Details on the enrichment/depletion measures are given in the Section “Strategy for the investigation of disordered regions and SNPs occurrence.”

**Figure 1:**
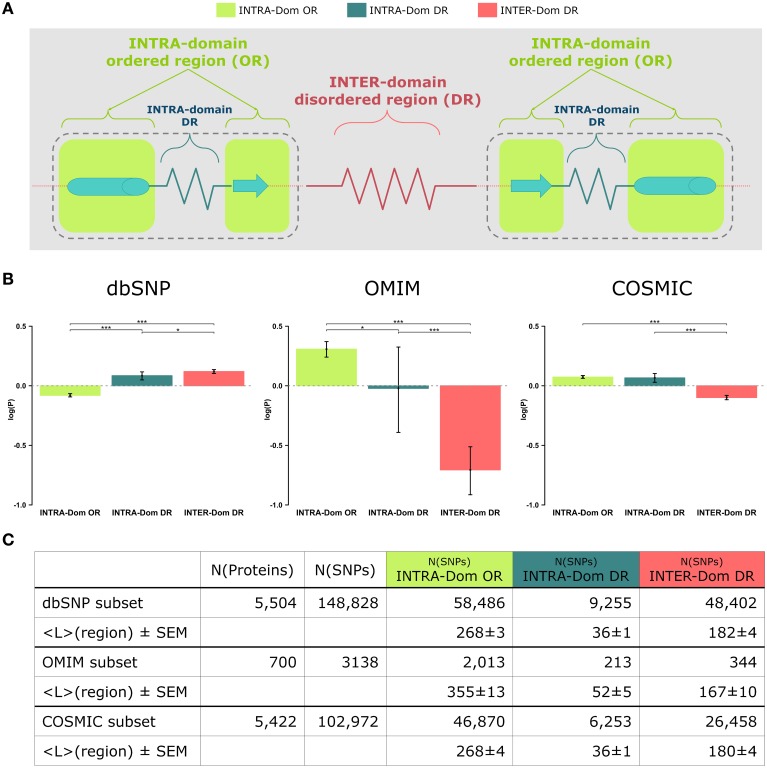
**Analyses of non-synonymous single nucleotide polymorphisms (SNPs) in intra-domain ordered regions, intra-domain disordered regions and inter-domain disordered regions**. **(A)** Scheme of protein regions. A protein contains (intra−)domain regions (dashed boundary line) and inter-domain regions. Domain regions contain ordered regions (INTRA-Dom ORs; light-green squares) and disordered regions (INTRA-Dom DRs; dark green zigzag line). Inter-domain regions are predominantly disordered (INTER-Dom DR; red zigzag line). **(B)** SNP frequency analysis. The propensity of SNPs P(SNP) to occur in each region was calculated using Equation 1. Average propensity values are reported as relative entropies log(P(SNP)). Error bars were estimated using bootstrap re-sampling with 10,000 replicates. Stars denote the alpha levels of the test statistics (^*^p < 0.05; ^***^p < 0.001). **(C)** Number of SNPs mapped onto different protein regions. The number of nsSNPs in each class and the average lengths of the protein regions are listed together with the standard error of the mean (SEM). The column “N(proteins)” contains the number of proteins selected for the study of a SNP class, while column “N(SNPs)” reports the total number of SNPs mapped onto the reference proteins.

The outcome of our analysis is presented Figure 1B and the barplots relative to each region are colored according to the scheme in Figure 1A. The results for dbSNP data are reported as a comparison of the observed human variation in the analyzed regions vs. the pathogenic mutations observed for OMIM and COSMIC data. To our knowledge, this is the first time that such a comparison is presented. The results have been statistically tested (see Strategy section) and the *p*-values of the comparison tests between the distributions are annotated with stars to show their significance (see Figure 1 legend for clarification).

The most striking difference amongst all data lies in the opposite behavior observed for INTER-domain disordered regions (INTER-Dom DRs, red) in dbSNP vs. OMIM and COSMIC data (enrichment vs. depletion, respectively). The reasons for such trend can be ascribed to the fact that common variations usually do not occur in structurally and functionally constrained regions but rather accumulate in disordered regions, particularly inter-domain ones. These are usually more flexible to allow the orientation of protein domains and binding multiplicity (Fong and Panchenko, 2010). Conversely, an opposite trend is observed for the INTRA-domain ordered regions (INTRA-Dom OR, light green) of dbSNP vs. the disease-related INTRA-Dom OR plots. For both the disease-related OMIM (Figure 1B, center) and COSMIC (Figure 1B, right) datasets, there is clear evidence that pathogenic mutations are enriched in ordered domain regions. These are the fragile sites that once mutated can cause a functional impairment of the protein either by destabilizing the fold (Studer et al., 2013), or by affecting structurally important regions for partner binding and consequent signaling activity (Yates and Sternberg, 2013). The enrichment in INTER-Dom disordered regions vs. INTRA-Dom ordered regions is particularly pronounced for the OMIM dataset, but also significantly important for the COSMIC data. The difference in the relative order/disorder populations of the two datasets might be related to the fact that mutations with Mendelian inheritance are potentially more harmful to the protein than some of the passenger mutations observed in cancer.

Our results support previous studies that compared differences in “natural” mutations from dbSNP and disease-associated OMIM data (De Beer et al., 2013). The difference in order vs. disorder propensities observed in our study is therefore an additional discriminant in evaluating the mutability of proteins.

### Examples of intra-domain mutations and effects on disorder occurrence

In a number of recent studies it has been reported that disordered regions harbor pathogenic mutations (Iakoucheva et al., 2002; Uversky et al., 2008; Babu et al., 2011; Hu et al., 2011; Pajkos et al., 2012; Vacic and Iakoucheva, 2012; Vacic et al., 2012). Some of these observations referred to SNPs in segments involved in D-O transitions, but as we observed a clear dearth of pathogenic mutations in INTER-domain disordered regions (INTER-Dom DRs), we decided to investigate the occurrence of SNPs in INTRA-Dom DRs in more detail. A particularly interesting case is the mutual effect of intra-domain pathogenic mutations and disorder observed within the domain, even at sites distant from the original mutation. We found such examples in BRAF and JAK2 kinases, which are involved in cancer pathologies (Vogelstein and Kinzler, 2004).

**Figure 2:**
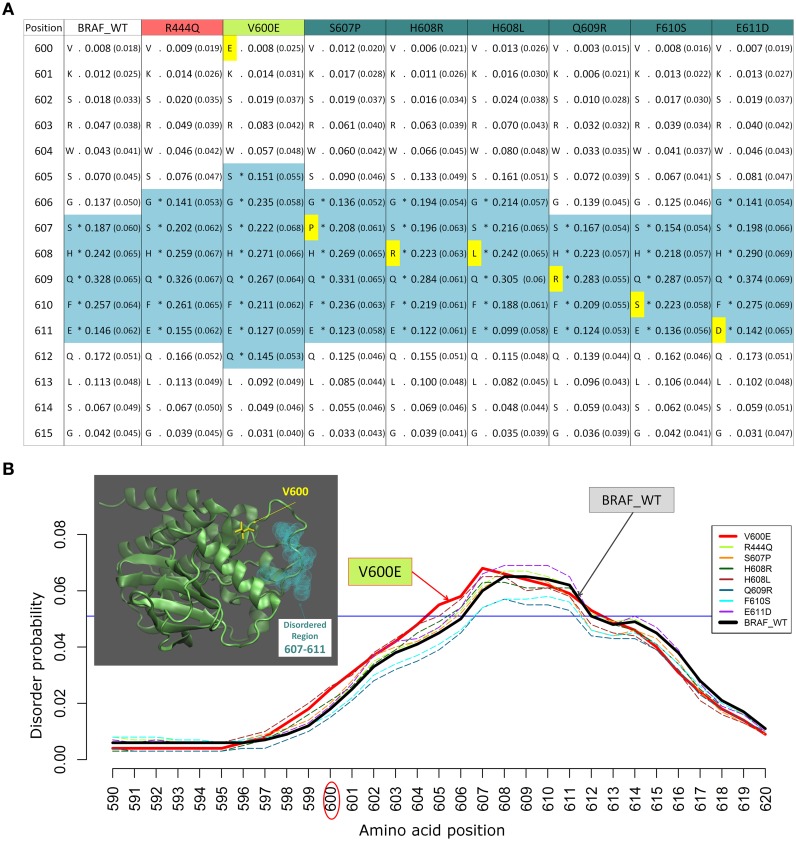
**Example of changes in disordered regions (DRs) conferred by SNPs in distant ordered regions**. **(A)** Disorder prediction by DISOPRED2 of wild type (WT) and mutated sequence segments (600–615) of BRAF. Each column is labeled with the specific SNP used for DR prediction and contains the confidence scores of the DISOPRED2 prediction involving raw scores of disorder probability and their filtered scores with parentheses. The residues in DRs are annotated with (^*^) asterisks and colored in blue. SNPs within the sequence segment 600–615 are colored in yellow. **(B)** Plot of the DISOPRED2 filtered confidence scores of the BRAF WT and mutated sequences. The predicted behavior of V600E (red line) is distinct from that of the BRAF WT sequence (thick black line). The horizontal blue line indicates 5% of filter threshold of the method. The inset shows the 3D structure of the BRAF kinase domain (4MNE_B, cyan cartoon), the location of residue V600 (yellow licorice) and the predicted disordered positions (light green spheres).

We previously studied the BRAF V600E mutation that destabilizes the inactive conformation of the BRAF kinase and consequently induces ERK activation (Satoh et al., 2012; Lu et al., 2013). The V600 residue is in a cluster of hydrophobic residues with Phe468, therefore the presence of a negative charge (residue E) will be disruptive for this cluster, resulting in destabilization of the inactive conformation. Interestingly, introducing the V600E mutation in the BRAF protein kinase domain increases the INTRA-Dom DRs prediction, as shown in the table (Figure 2A) and the plot (Figure 2B). By running the DISOPRED2 predictor for the V600E mutant, one can observe an increase in the span of the predicted disordered region found in a distal site (607–611). Notably, the predicted disorder region span was not affected by mutations found within the INTRA-Dom DRs (yellow residues in Figure 2A for BRAF). These findings suggest that, besides destabilizing the hydrophobic cluster, the V-E substitution in the kinase domain (Pkinase_Tyr(PF07714)) might also have an effect on the INTRA-Dom disorder content by unwinding the downstream loop, as shown in the wild type 3D structure (4MNE_B) (Haling et al., 2014) (Figure 2B and Figure S1). This could in turn affect the ligand-binding region (structure 4WO5_A) (Thevakumaran et al., 2015), with a possible impact on the binding affinity.

The mutation V600E has been studied in detail by sophisticated enhanced sampling methods (Marino et al., 2015) and one of the main consequences of the pathogenic variant highlighted in this study is reflected the enhancement of the active-to-inactive state barrier and the increased flexibility (disorder) of the activation loop (region 602–612). These combined effects result in keeping the kinase in an active state and therefore favor phosphorylation to occur. This study supports the idea that an accurate descriptions of the structure, dynamics, and energetics of the protein and its mutated states are necessary to extract molecular fingerprints that rationalize the impact of pathogenic vs. commonly occurring mutations. Interestingly, in recent times the tendency of BRAF in adopting permanently an active state not detectable by current structure has been highlighted as one of the paradigmatic cases for which the currently adopted strategies for structure-based drug discovery may be ineffective (Holderfield et al., 2014).

Long-range effects of mutations on domain-disorder content are partially observed also for the V617F SNP of the JAK2 kinase, a mutation mostly observed in leukaemias. Our predictions indicate that this mutation leads to an extension of the INTRA-Dom DRs, (Bandaranayake et al., 2012) as shown in the table (Figure S2D) and the plot (Figure S2E).

The changes of disorder probability between the wild type sequences (BRAF and JAK2) and those with the cancer driver SNPs (V600E and V617F, respectively) have been predicted by five different methods which include highly ranked methods in CASP10 (Monastyrskyy et al., 2014) such as DISOPRED3, PrDOS, Biomine_MFDp and a recent method using backbone dynamics, DynaMine (Cilia et al., 2014) (Figures S2, S4). The results do not show a strict consensus in the boundaries and in the absolute differences of disorder content, this can be ascribed to the different algorithms used. However, most of the methods predict an increase of the disorder probability in the mutation distal regions we observe for BRAF that the cancer driver mutation is at the periphery of the kinase binding site and in an ordered region, while the non-driver mutations mostly occur in the disordered regions. The two locations seem to be correlated in the sense that the observed change in the driver mutation alters the disorder content of the other mutation loci. This may be ascribed to correlated dynamical couplings between disordered and ordered regions within the same protein domain that may lead to an enrichment of pathogenic variants in flexible and less structured regions. This long-range coupling is an indirect and probably down-tuned mutational effect on the protein function, which may result in a higher acceptance of the mutation in these regions.

## Strategy for the investigation of disordered regions and SNP frequencies

### Data set preparation

A data set of human proteins, using UniProt accession identifiers as reference, was generated by mapping SNPs onto experimentally resolved 3D structures of proteins. Native and homologous structures were identified by running NCBI-BLAST (version 2.2.29+) (Camacho et al., 2009) against the PDB sequence library. Homologues were accepted above the 30% sequence identity threshold. Non-synonymous SNPs were retrieved from the dbSNP database (build 141) (Sherry et al., 2001), germ-line disease-related SNPs were extracted from the “Online Mendelian Inheritance in Man” (OMIM) database (version July 2014) (Hamosh et al., 2005) and somatic cancer-related SNPs were taken from the “Catalog of Somatic Mutations in Cancer” (COSMIC) database (version July 2014) (Forbes et al., 2011). Only the proteins having a native/homologous structure and SNP information were selected, yielding a reference data set comprising 5587 proteins.

### Definition of protein domains and disordered regions

The selected proteins were assigned with domain definitions and disordered region predictions. For each protein sequence of the reference data set as query, the HMM sequence aligner HMMER3 (Finn et al., 2011) was used to search against the Pfam domain sequence library (Pfam-A.hmm version 26.0) (Punta et al., 2012) and to assign the matched PFAM domain definition to the query protein, given the alignment *E*-value was smaller than 1e-3. Disordered regions of the selected proteins were predicted using the DISOPRED program (Ward et al., 2004). The combination of domain definitions and disordered region predictions leads to three distinct regional classes (Figure 1A): (1) intra-domain ordered region (INTRA-Dom OR), (2) intra-domain disordered region (INTRA-Dom DR), and (3) inter-domain disordered region (INTER-Dom DR).

### SNPs enrichment analysis

We computed the regional enrichment/depletion of SNPs as propensities P(SNPregion) by normalizing the relative regional frequency with the relative frequency over the total protein length (Equation 1).

(1) $$\begin{array}{l} P\left({SNP}_{region}\right)=\frac{\left({N\left(SNPs\right)}_{region}∕{length}_{region}\right)}{\left({N\left(SNPs\right)}_{protein}∕{length}_{protein}\right)} \end{array}$$

These propensities are plotted in Figure 1B as relative entropies log(P(SNPregion)). A relative entropy of zero indicates a regional frequency equal to the background frequency (denominator), positive values indicate relative enrichment and negative values correspond to relative depletion. All SNPs (from dbSNP, OMIM, and COSMIC) were mapped onto the protein sequences: 5504 of 5587 proteins were mapped with SNPs from dbSNP, 700 of 5587 with SNPs from OMIM and 5422 of 5587 with SNPs from COSMIC. SNPs from each database were further classified into different classes by mapping their positions onto the corresponding protein regions (INTRA-Dom OR SNPs, INTRA-Dom DR SNPs, and INTER-Dom DR SNPs). The number of SNPs in the different classes and the mean lengths of the regions are given in Figure 1C.

### Statistical evaluation

To obtain an estimate of the uncertainty associated with the propensity calculations and to reduce biases incurred by the protein selection procedure, we used a bootstrapping method (R function *boot*()) to create random re-sampled subsets of the reference data set. 10,000 independent subsets were generated of the SNPs propensities within each pre-defined protein regional class (INTRA-Dom OR SNPs, INTRA-Dom DR SNPs, and INTER-Dom DR SNPs) and the mean of each subset was computed. The distributions of the resampled means are by normally distributed, as expected (Figure S4). Confidence intervals at the 95% level were calculated from the bootstrap distributions. The statistical significance of differences between propensity distributions was calculated by Student's *t*-test on the confidence intervals (Wolfe and Hanley, 2002). The statistical analyses were performed using R (R Core Team, 2014).

## Conclusions and perspectives

We performed a large-scale statistical analysis of the relationship between protein disorder and disease-related mutations. We report that both genetic-disease variants from OMIM and cancer-related SNPs from COSMIC are depleted in disordered regions compared to common human variation. This is in line with the fact that mutations in highly constrained regions of the protein are more likely to be disruptive or deleterious. This is why mutations in ordered states of proteins (domains, ligand-binding sites, PPI sites) have been investigated quite in detail in the last years.

We offer here a starting and objective point to discriminate between completely ordered regions, disordered regions occurring in ordered domains, and inter domain predicted disordered segments. We observe and quantify the result of the mapping of available SNPs data onto a large set of human proteins and their close homologs. From this study a number of interesting cases can be extracted for functional validation and close investigation of the dynamical role played by the disorder content.

New perspectives in the field can be explored from this starting point, as the more complicate cases in which flexibility and/or disorder play a direct role in the protein function have not yet been fully elucidated. Particularly complex are the cases where flexible residues modulate protein binding and promiscuity (Fornili et al., 2013) and disorder-to-order causing mutations (Vacic and Iakoucheva, 2012; Dunker et al., 2015). These more “dynamically” driven processes are difficult to parametrise and the restraints playing a role in selecting the actual functional states are not always quantifiable. At this purpose, systematic studies collecting critical examples of experimentally proved correlations between flexibility, presence of disordered states, D-O transitions, and functional studies are needed in the field for the benchmarking and validation of predictive tools for the impact of pathogenic variation on proteins and their partners. Most recent development in disorder prediction methods exploits successfully the mutual interplay between backbone and side-chain dynamics (Cilia et al., 2014; Kosciolek and Jones, 2015).

Nevertheless, more sophisticated methods are needed to quantify these observations, like large-scale molecular simulations, [so far performed for isolated cases Vacic et al., 2012; Marino et al., 2015] and measurements of conformational signal transduction within protein structures (Pandini et al., 2012). The correlated dynamical couplings between disordered and ordered regions may be exploited in the design of drugs targeting distal sites from the dominant mutation, and by fine-tuning the effects on the overall protein function. Additionally, the possibility to predict the “allosteric” modulation of mutations occurring in regions with a different level of order/disorder and possibly correlated with the same or different pathogenic manifestation can open new avenues to investigate the underlying molecular mechanisms and rectify current strategies for drug-discovery.

### Conflict of interest statement

The authors declare that the research was conducted in the absence of any commercial or financial relationships that could be construed as a potential conflict of interest.
