# Supplementary material for: Anatomy of protein disorder, flexibility and disease-related mutations
Source: Front Mol Biosci. 2015 Aug 12;2:47. doi: 10.3389/fmolb.2015.00047 (PMC4532925; doi:10.3389/fmolb.2015.00047)
Supplement: Supplementary file 1 [file DataSheet1.PDF]

Supplementary Material

Anatomy of protein disorder, flexibility and disease-related mutations.

Hui-Chun Lu[1], Sun Sook Chung[1,2], Arianna Fornili[1,3], Franca Fraternali[1]*

[1]Randall Division of Cell and Molecular Biophysics, King's College London, London, United Kingdom
[2]Department of Haematological Medicine, King's College London, London, United Kingdom
[3]School of Biological and Chemical Sciences, Queen Mary University of London, London, United Kingdom

*** Correspondence:** Franca Fraternali, Randall Division of Cell and Molecular Biophysics, King's College London, London, SE1 1UL, United Kingdom
franca.fraternali@kcl.ac.uk

1. Supplementary Figures

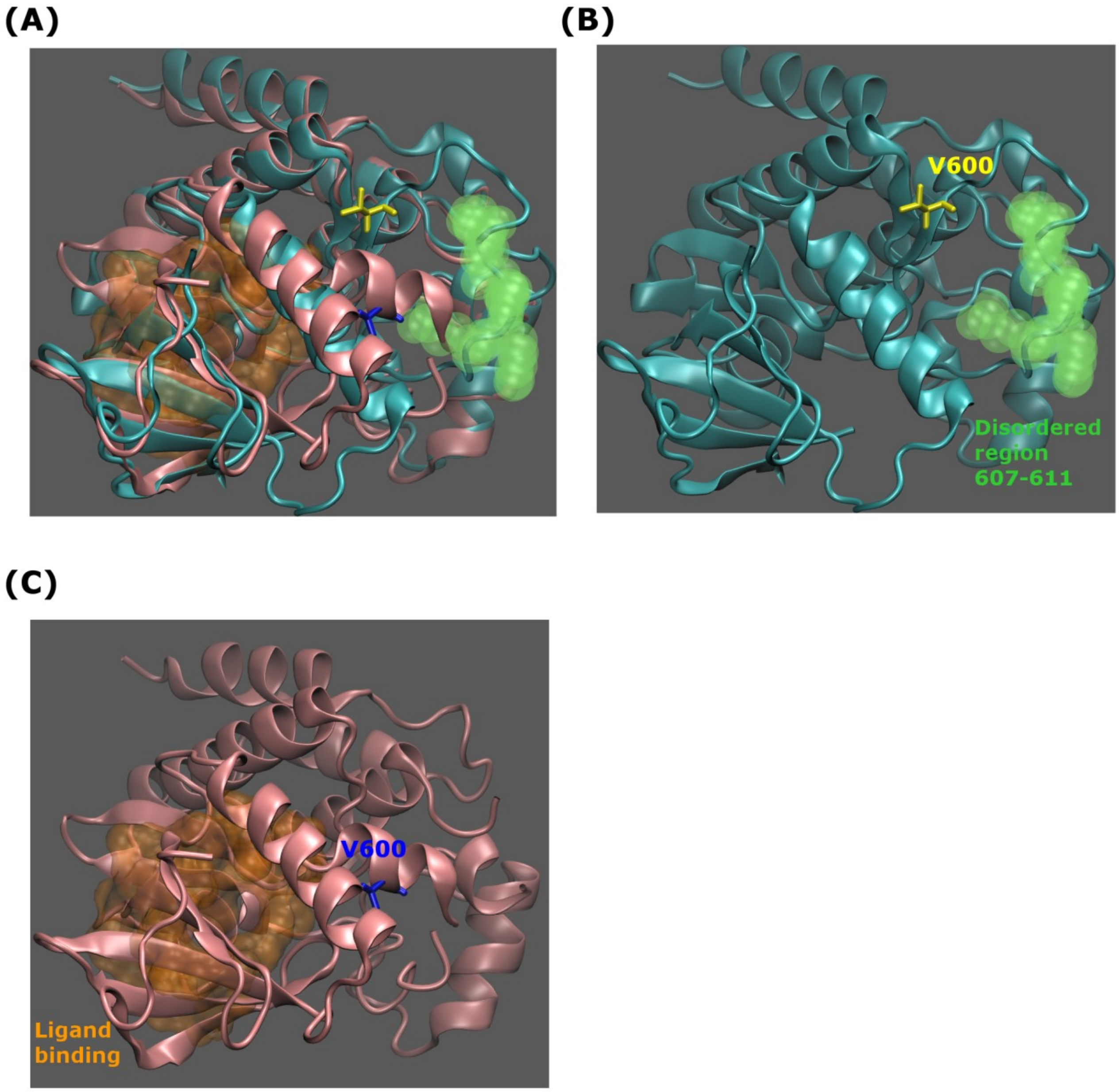

Supplementary Figure 1. Cartoon representations of the 3D structures of the BRAF kinase domain shown in Figure 2. (A) Superimposition of the 3D structures of BRAF kinase domain from two different PDB files: 4MNE_B (cyan cartoon) and 4WO5_A (pink cartoon). (B) The structure of BRAF kinase domain (4MNE_B) indicating the location of residue V600 (yellow licorice) and the predicted regions (light green spheres). (C) The structure of BRAF kinase domain (4WO5_A) indicating the location of residue V600 (blue licorice) and the ligand binding regions (orange spheres) as described in the original study of the structure model (Thevakumaran et al., 2015).

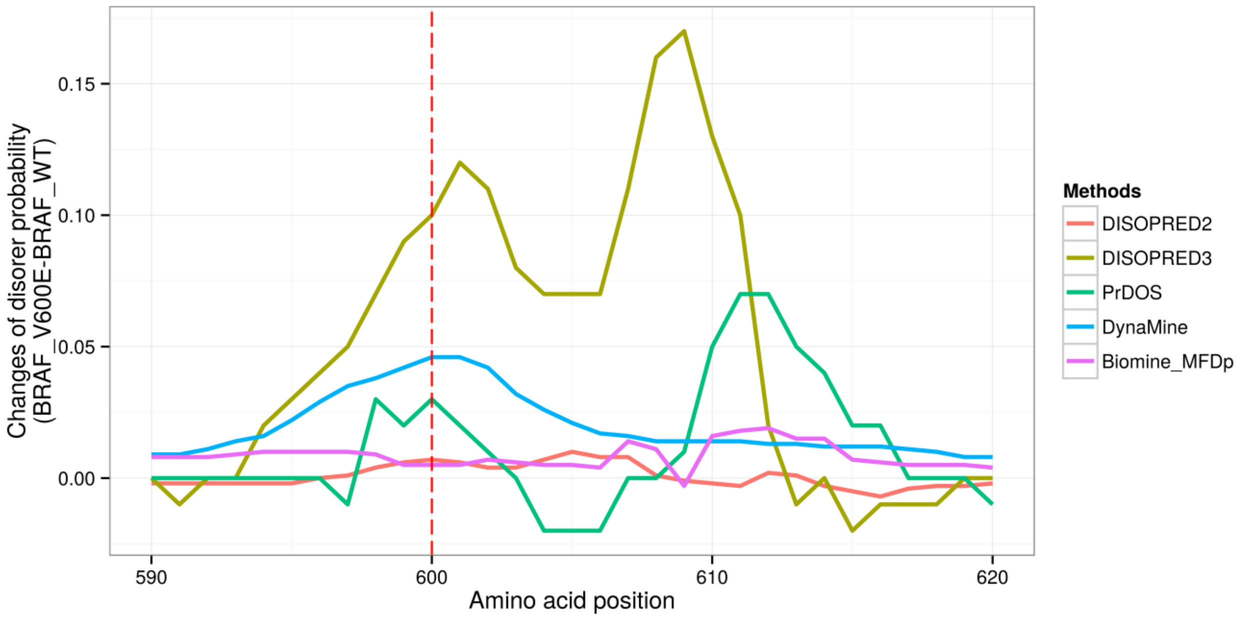

Supplementary Figure 2. Disorder probability change between the sequence of BRAF WT and the mutated one BRAF_V600E as predicted by the methods: DISOPRED2, DISOPRED3, PrDOS, DynaMine and Biomine_MFDp. Plots comparing changes of disorder probability between BRAF_WT and BRAF_V600E colored according to the tested methods. The red dotted line indicates the position of the mutation in the sequence numbering.

(A)

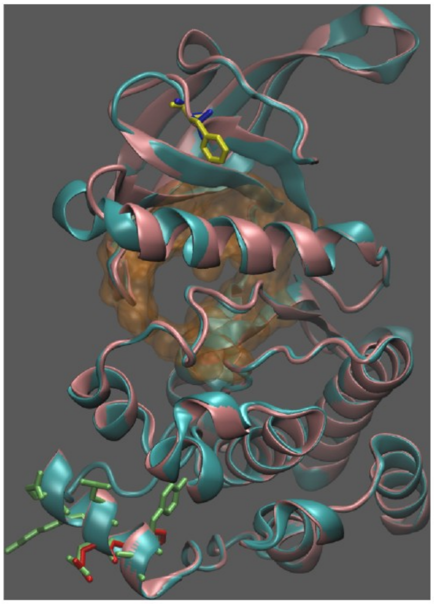

(B)

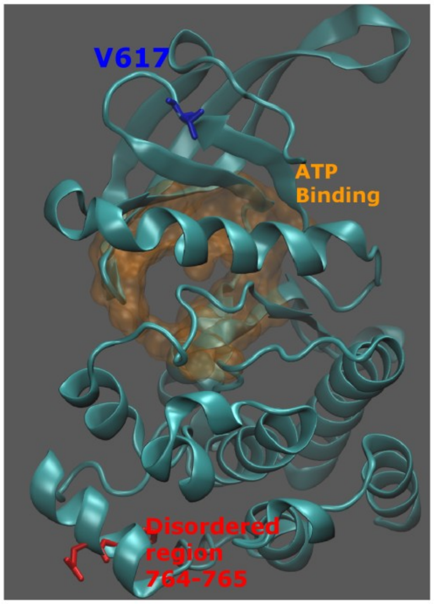

(C)

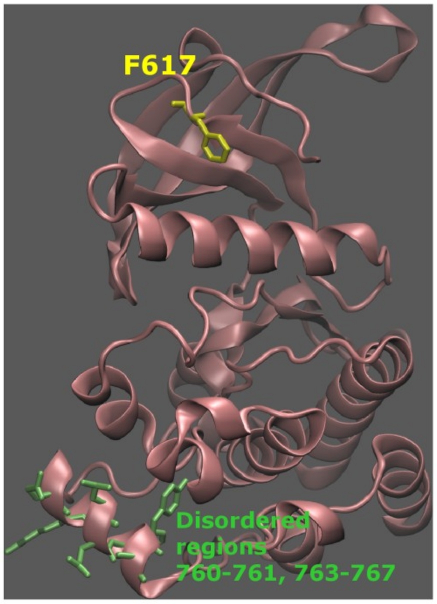

(D)

Position	JAK2_WT	V617F
759	S . 0.235 (0.040)	S . 0.234 (0.044)
760	Q . 0.104 (0.049)	Q * 0.124 (0.054)
761	R . 0.080 (0.046)	R * 0.094 (0.052)
762	K . 0.091 (0.045)	K . 0.111 (0.052)
763	L . 0.118 (0.049)	L * 0.156 (0.056)
764	Q * 0.066 (0.053)	Q * 0.083 (0.061)
765	F * 0.113 (0.053)	F * 0.149 (0.061)
766	Y . 0.084 (0.047)	Y * 0.119 (0.055)
767	E . 0.098 (0.043)	E * 0.114 (0.053)
768	D . 0.087 (0.041)	D . 0.114 (0.048)

(E)

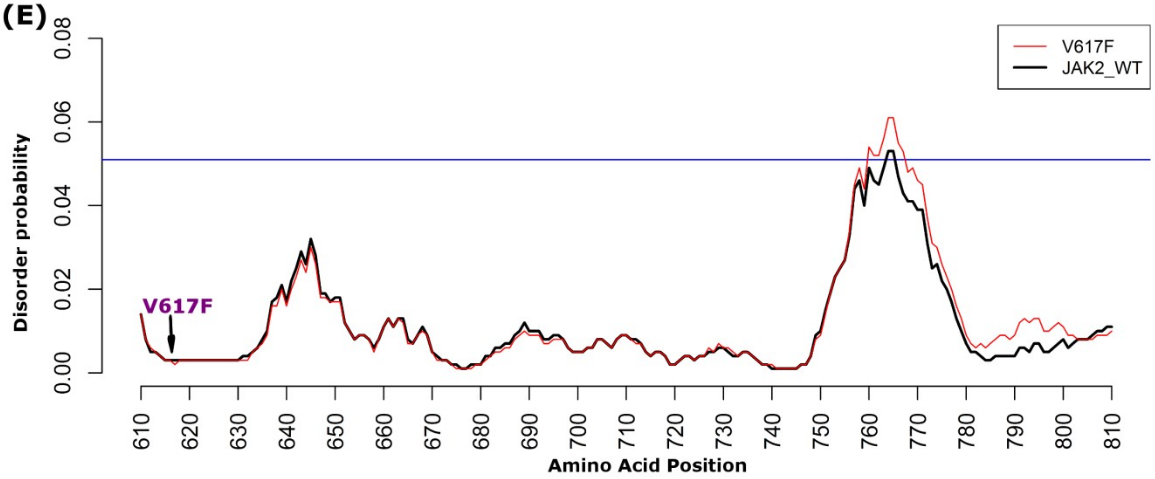

Supplementary Figure 3. Example of changes in disordered regions for the JAK2 Pseudokinase domain. (A) Superimposition of the 3D structures of the JAK2 Pseudokinase domain from different PDB files: 4FVQ_A (cyan cartoon) and 4FVR_A (pink cartoon). (B) The structure of JAK2 Pseudokinase domain (4FVQ_A) indicating the location of residue V617 (blue licorice) and the predicted disordered regions (red licorice). (C) The structure of JAK2 Pseudokinase domain with the variant of V617F (4FVR_A) with the location of residue F617 (yellow licorice) and the predicted disordered regions (light green licorice). (D) Disorder prediction by DISOPRED2 of WT and mutated sequence residues (610-810) of JAK2. The table shows the confidence scores of the DISOPRED2 prediction for the wild type and the sequence with V617F. The residues in DRs are annotated with asterisks (*) and coloured in blue. (E) Plot of the DISOPRED2 filtered confidence scores for the JAK2 WT and a mutated sequence. The plot of V617F (red line) shows a slight increase in the disordered region when compared to the JAK2 WT (black line).

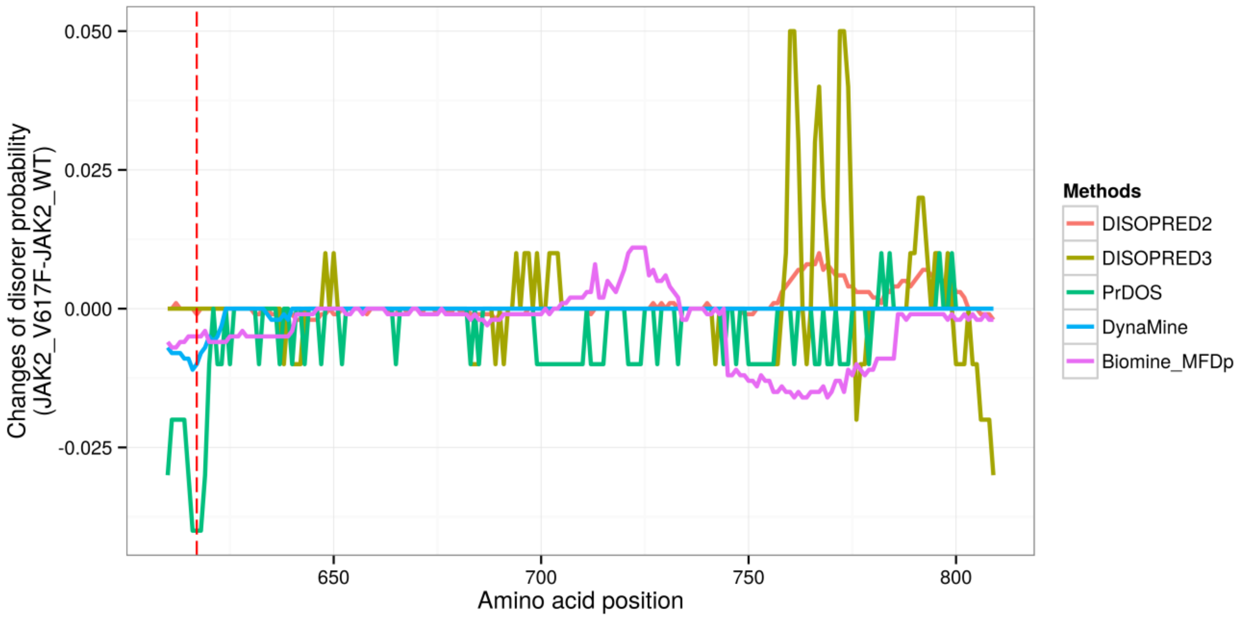

Supplementary Figure 4. Disorder probability changes between the sequenceof JAK2 WT and the mutated one JAK2_V617F as predicted by the methods: DISOPRED2, DISOPRED3, PrDOS, DynaMine and Biomine_MFDp. Plots comparing changes of disorder probability between JAK2_WT and JAK2_V17F colored according to the tested methods. The red dotted line indicates the position of the mutation in the sequence numbering.

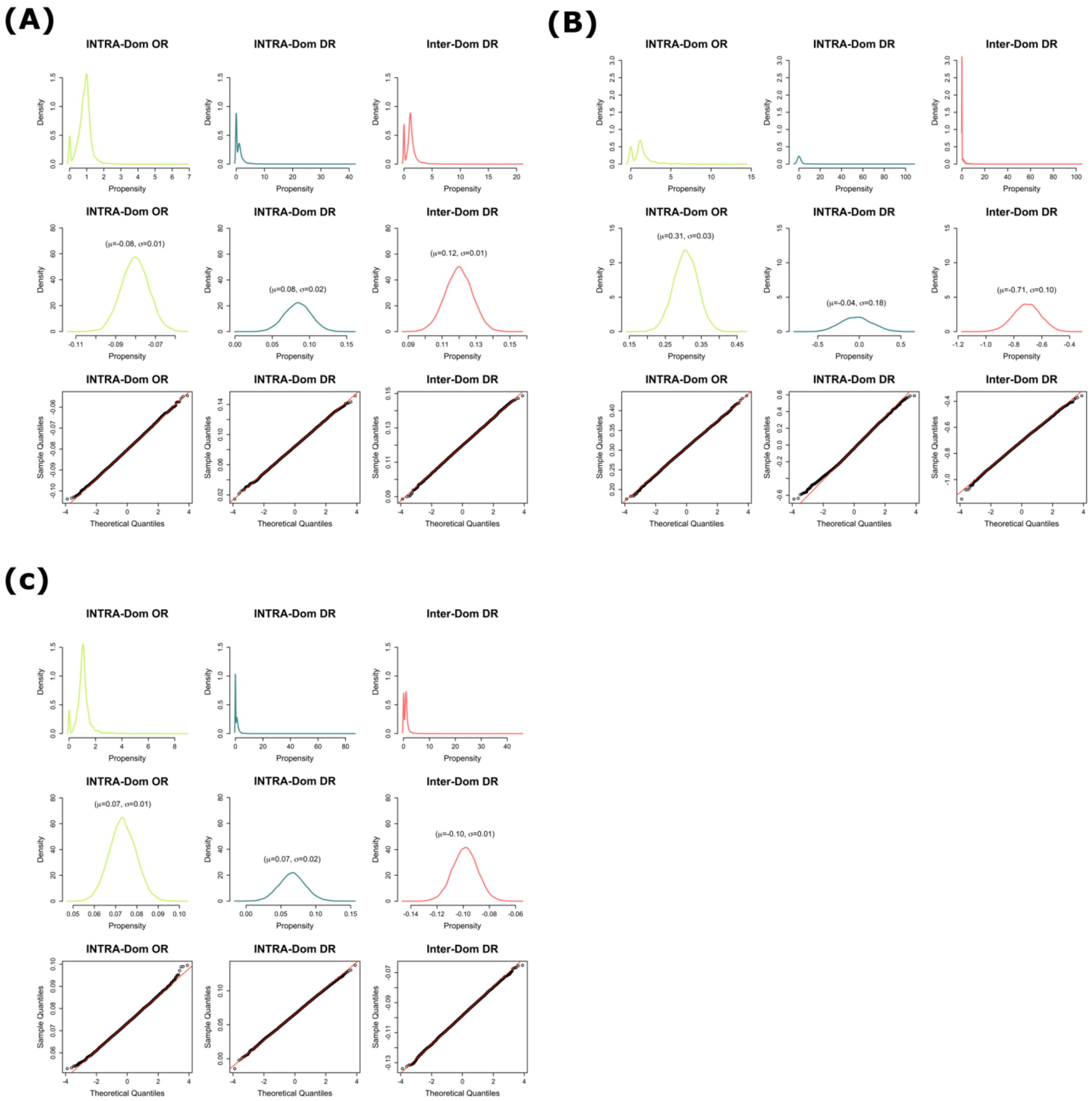

Supplementary Figure 5. Distribution of the propensities of SNPs from (A) dbSNP, (B) OMIM and (C) COSMIC separated into structural regions: INTRA-Dom OR, INTRA-Dom DR and Inter-Dom DR. The distributions of sample data, resampled data, and Q-Q plots are shown. For each database, the top row shows the distributions of the propensities of SNPs occurring in the different structural regions analysed. The middle row shows the distributions of the means of resampled subsets (bootstrapping). The mean and standard deviation of each distribution are given. The bottom row shows Q-Q plots in which the resampled distributions are plotted over the theoretical normal distributions with the given mean and standard deviation to illustrate the normality of the resampled means.
